# P-2008. Chronic Conditions as Risk Factors for COVID-19–Associated Hospitalization Among Adults, 2022-2023

**DOI:** 10.1093/ofid/ofae631.2165

**Published:** 2025-01-29

**Authors:** Sarah Hamid, Gordana Derado, Huong Pham, Rebecca C Woodruff, Darpun Sachdev, Breanna Kawasaki, James Meek, Lucy S Witt, Patricia A Ryan, Sue Kim, Erica Bye, Jennifer Akpo, Jemma Rowlands, Erin Licherdell, Eli Shiltz, Melissa Sutton, Keipp Talbot, Andrea Price, Fiona P Havers, Christopher Taylor

**Affiliations:** Centers for Disease Control and Prevention, New York, New York; CDC, Atlanta, Georgia; Centers for Disease Control and Prevention, New York, New York; Centers for Disease Control and Prevention, New York, New York; California Department of Public Health, Richmond, California; Colorado Department of Public Health and Enviornment, Denver, Colorado; Connecticut Emerging Infections Program, Yale School of Public Health, New Haven, Connecticut; Emory University, Atlanta, Georgia; Maryland Department of Health, Baltimore, Maryland; Michigan Department of Health and Human Services, Lansing, Michigan; Minnesota Department of Health, Saint Paul, Minnesota; New Mexico Department of Health, Santa Fe, New Mexico; New York State Department of Health, Albany, New York; University of Rochester Medical Center, Rochester, New York; Ohio Department of Health, Columbus, Ohio; Oregon Health Authority, Portland, Oregon; Vanderbilt University Medical Center, Nashville, Tennessee; Salt Lake County Health Department, SLC, Utah; CDC, Coronavirus and Other Respiratory Viruses Division, Atlanta, Georgia; CDC, Atlanta, Georgia

## Abstract

**Background:**

Risk factors for COVID-19-associated hospitalizations were identified early in the pandemic. Since then, population immunity to SARS-CoV-2 has increased substantially and hospitalizations for COVID-19 have decreased. Identifying populations at greatest risk of COVID-19–associated hospitalization can help guide prevention and treatment efforts. We aimed to update information on chronic conditions as risk factors for COVID-19-associated hospitalization.

**Figure.**
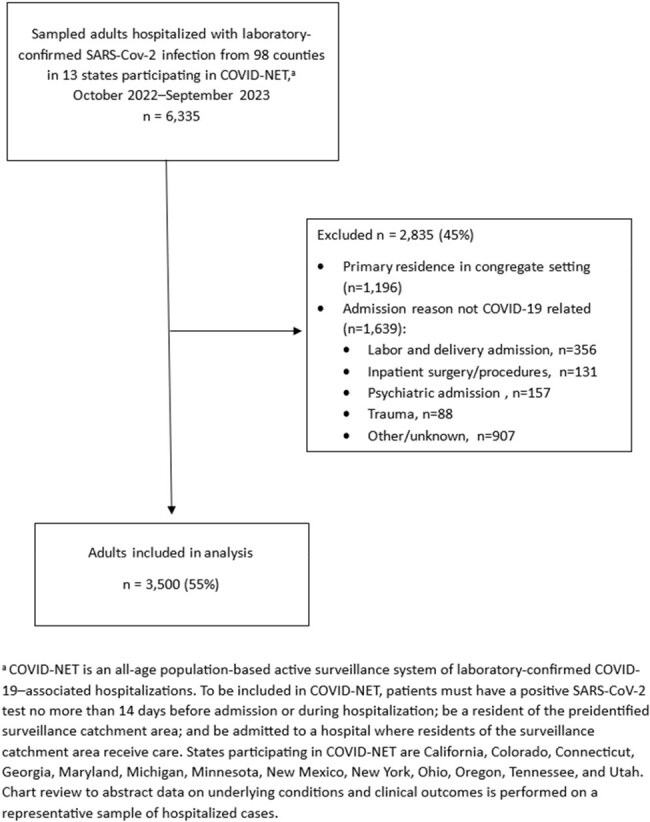
Flow diagram of participant selection into analytic population, Coronavirus Disease 2019-Associated Hospitalization Surveillance Network (COVID-NET), 2 October 2022–30 September 2023.

**Methods:**

We compared hospitalization rates in adults aged ≥ 18 years with vs without 9 chronic conditions during 2 October 2022–30 September 2023. Rate numerators were COVID-19–associated hospitalization counts from the COVID-19–Associated Hospitalization Surveillance Network, a population-based active surveillance system with a 98-county catchment area in 13 states. Rate denominators were counts of adults with and without chronic conditions in the catchment area, estimated with US census county-level population data and state-level estimates of the adult population with and without select chronic conditions from the 2022 Behavioral Risk Factor Surveillance System. Rate ratios and 95% confidence intervals were estimated using Poisson regression with Monte Carlo simulation, adjusting for age group, sex, and race/ethnicity.

**Figure:**
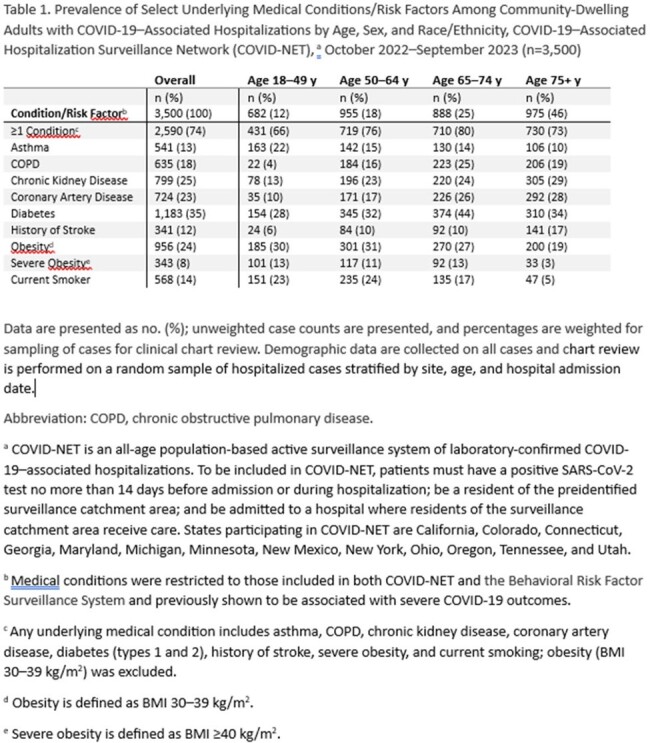

**Results:**

Among 3,500 hospitalized adults, 74% had ≥ 1 chronic condition (Figure, Table 1). Hospitalization rates were higher among those with ≥ 1 chronic condition (vs none); adjusted rate ratios (aRR) ranged from 2.2 in adults aged ≥ 75 years to 4.6 in adults aged 18-49 years (Table 2). Among adults overall, chronic kidney disease was associated with the greatest risk of COVID-19–associated hospitalization (aRR: 4.5), followed by diabetes (2.2), history of stroke (2.0), severe obesity (2.0), coronary artery disease (1.9), COPD (1.9), smoking (1.6), and asthma (1.5); results varied by age and generally attenuated with older age (Table 2). Adjusting for number of chronic conditions, hospitalization rates were higher for adults aged ≥ 75 years relative to 18–49 years (18.5) (Table 3).

**Figure:**
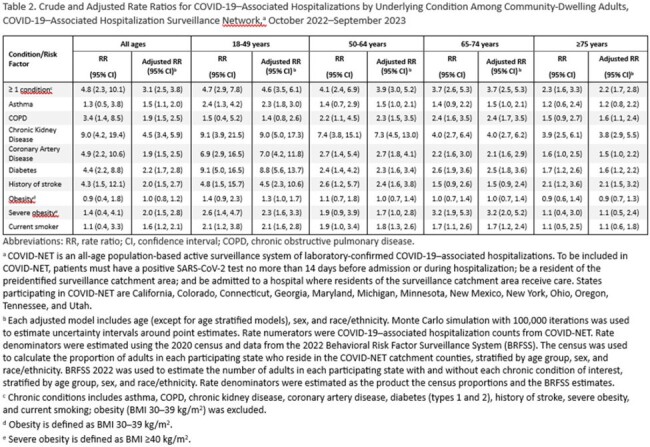

**Conclusion:**

Of 9 chronic conditions, 8 were associated with increased risk of COVID-19–associated hospitalization with risk varying by condition and age group. Older age remains the strongest risk factor.

**Figure:**
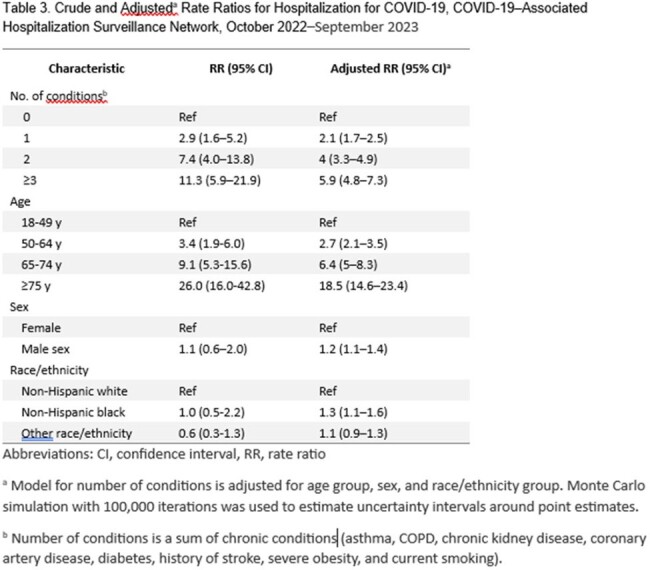

**Disclosures:**

Sue Kim, MPH, Council of State and Territorial Epidemiologists: Grant/Research Support

